# Risk of hepatitis B reactivation in COVID-19 patients receiving corticosteroid therapy: A retrospective cohort study

**DOI:** 10.1097/MD.0000000000050373

**Published:** 2026-08-28

**Authors:** Hatun Öztürk Çerik, Özlem Aldemir, Bilge Katirci, Burcu Demirhan Kapicioğlu, Zeynep Banu Ramazanoğlu, Arzu Altunçekiç Yildirim

**Affiliations:** a Department of Infectious Diseases and Clinical Microbiology, Ordu University School of Medicine, Ordu, Türkiye; b Department of Infectious Diseases and Clinical Microbiology, Ministry of Health Sivas Numune Hospital, Sivas, Türkiye.

**Keywords:** Antiviral prophylaxis, Corticosteroids, COVID-19, Hepatitis B, Reactivation

## Abstract

COVID-19 and HBV are significant public health issues, and managing their co-infection remains unclear. Corticosteroid therapy improves survival in severe COVID-19 but may increase the risk of HBV reactivation (HBVr). Evidence regarding the incidence and outcomes of HBVr in COVID-19 patients remains limited. This study aimed to investigate the frequency, determinants, and clinical consequences of HBVr among COVID-19 patients with current or past HBV infection treated with corticosteroids. This retrospective study evaluated adult patients with COVID-19 and evidence of HBV infection hospitalized between March 2020 and January 2022 at a tertiary center, where 13,673 individuals were screened during the study period. Demographics, corticosteroid use, laboratory findings, HBV DNA results, and outcomes were analyzed. HBVr was defined according to the criteria outlined in current international guidelines. Out of 13,673 hospitalized COVID-19 patients, 145 (125 HBsAg-positive and 20;anti-HBcIgG-positive) were identified as having HBV exposure. Corticosteroids were administered to 75 patients (51.7%) with a median cumulative dose of 520 (240–1400) mg and duration of 6 (4–10) days. HBVr occurred in 7 patients, all HBsAg-positive; 2 developed hepatic failure and died. Patients with HBVr had significantly higher cumulative corticosteroid doses (p=.021) and longer durations of therapy (p=.007) compared with those without reactivation. Five patients received nucleos(t)ide analogues with subsequent viral suppression. COVID-19 patients with chronic HBV infection, particularly those receiving corticosteroids, are at increased risk of HBVr and poor outcomes. Based on our findings, HBV screening and consideration of antiviral prophylaxis should be routine prior to corticosteroid therapy, particularly in regions with intermediate-to-high HBV prevalence, since HBVr may occur even with short-term corticosteroid exposure.

## 1. Introduction

Coronavirus disease 2019 (COVID-19), caused by severe acute respiratory syndrome coronavirus 2 (SARS-CoV-2), has posed an unprecedented global health challenge. While most patients recover, approximately 20% develop severe complications such as acute respiratory distress syndrome (ARDS), multi-organ involvement, and the need for intensive care support.^[1]^ Corticosteroids have been shown to improve survival in severe COVID-19, and their use has become standard of care.^[2]^ Chronic hepatitis B virus (HBV) infection remains another major global health burden, affecting an estimated 257 million individuals worldwide.^[3]^ The COVID-19 pandemic introduced new challenges for patients with HBV, particularly due to the widespread use of immunosuppressive therapies such as corticosteroids, interleukin (IL)-6 and IL-1 receptor antagonists. While effective in controlling the hyperinflammatory response of severe COVID-19, these agents may also increase the risk of HBV reactivation (HBVr).^[4]^ Although early reports suggested a potential risk of HBV reactivation following the use of corticosteroids or anti-cytokine agents in COVID-19 patients with HBV infection, these observations were primarily based on expert opinion and limited evidence rather than cohort data.^[5]^

HBVr is defined as the reappearance or increase of HBV-DNA in patients with chronic or resolved infection, potentially leading to hepatitis flares, hepatic failure, or death.^[6]^ The risk of HBVr depends on both the host’s HBV serostatus and the type, dose, and duration of immunosuppression. Although high-dose corticosteroid regimens have been considered a potential risk factor, particularly in HBsAg-positive patients, current evidence to support this association remains limited.^[7,8]^ Nevertheless, HBV reactivation has been extensively reported in patients receiving chemotherapy or other immunosuppressive therapies, but data on its occurrence during COVID-19 treatment remain insufficient. Reports are largely confined to small series or case reports, and the incidence, risk factors, and outcomes of HBVr in this setting are not well established. Clinical presentations of HBV reactivation vary from subclinical asymptomatic status to fatal fulminant hepatitis.

## 2. Objectives

Therefore, this study aimed to investigate the incidence, risk, and clinical impact of HBV reactivation among COVID-19 patients with current or past HBV infection who received corticosteroid therapy. Our findings may help inform screening and prophylaxis strategies in this high-risk population.

## 3. Material and methods

### 3.1. Study design and patients

This retrospective study included adult patients hospitalized with COVID-19 at a tertiary center between March 1, 2020, and January 1, 2022. Patients with evidence of HBV infection, defined as positive hepatitis B surface antigen (HBsAg) and/or anti-hepatitis B core antibody (anti-HBc IgG), were eligible. Patients without follow-up data after discharge were excluded.

### 3.2. Data collection

Clinical and laboratory data were retrieved from electronic hospital records and outpatient follow-up visits. Demographic characteristics, comorbidities, and COVID-19 outcomes (including mortality) were recorded. The total cumulative dose and duration of systemic corticosteroid therapy were documented. Laboratory parameters included white blood cell count, platelet count, alanine aminotransferase (ALT), aspartate aminotransferase (AST), international normalized ratio (INR), HBsAg, anti-HBs, anti-HBc IgG, and HBV-DNA levels.

### 3.3. Definition of HBV reactivation

HBVr was defined according to international guidelines (AASLD 2018, AGA 2015, APASL 2021).^[7,9,10]^ In HBsAg-positive patients, reactivation was defined as an increase in HBV-DNA ≥ 2 log from baseline or HBV-DNA > 10,000 IU/mL. In HBsAg-negative/anti-HBc IgG-positive patients, HBVr was defined as reappearance of HBV-DNA or seroreversion to HBsAg positivity. Hepatitis flare was defined as ALT ≥ 3 × baseline or > 100 IU/L. The diagnostic criteria for HBVr according to AASLD-2018, AGA-2015, and APASL-2021 guidelines are summarized in Table 1.

**Table 1:** Definitions of HBV reactivation according to guidelines.

Patient Group		AGA 2015	APASL 2021	AASLD 2018
**HBsAg-positive**	**HBV DNA** **baseline unknow**	Not clearly defined	Not clearly defined	HBV DNA ≥ 10,000 IU/mL
	**HBV DNA** **Baseline Previously Undetectable**	Detectable DNA	≥100 IU/mL	≥3 log (1000 IU/mL)
	**HBV DNA** **Baseline Previously Detectable**	≥1 log (10 times) ↑	≥2 log (100 times) ↑	≥2 log (100 times) ↑
**HBsAg-negative** **Anti-HBc-positive**		Detectable HBV-DNA or HBsAg seroreversion	Detectable HBV-DNA or HBsAg seroreversion	Detectable HBV-DNA or HBsAg seroreversion

AASLD = American Association for the Study of Liver Diseases,^[10]^ AGA = American Gastroenterological Association Institute,^[6]^ APASL = Asian-Pacific Association for the Study of the Liver.^[9]^

### 3.4. Statistical analysis

Categorical variables were expressed as numbers and percentages, and continuous variables as mean ± standard deviation or median with interquartile range (IQR), depending on distribution. Normality was tested with the Kolmogorov–Smirnov test. Continuous variables were compared using Student *t* test or Mann–Whitney *U* test; categorical variables were compared with chi-square test. A *P*-value < .05 was considered statistically significant. Statistical analyses were performed using SPSS version 22.0 (IBM Corp., Armonk, NY, USA).

### 3.5. Ethical considerations

The study protocol was approved by the Clinical Research Ethics Committee of Sivas Cumhuriyet University (Approval number: CUTF KAEK 2022–05/08). Due to the retrospective design of the study, formal informed consent was waived in accordance with local regulations and institutional guidelines. The study was conducted in accordance with the 1964 Declaration of Helsinki and its later amendments.

## 4. Results

During the study period, 13,673 patients were hospitalized with COVID-19, of whom 145 (1.0%) had evidence of HBV infection or exposure. Among these, 125 (86.2%) were HBsAg-positive and 20 (13.8%) had resolved/occult infection (HBsAg-negative/anti-HBc IgG-positive). Among the 125 HBsAg-positive patients, pre-COVID baseline HBV-DNA data were available for 100 patients, while 25 patients had no baseline HBV-DNA measurement. Among those with available data, HBV-DNA was undetectable in 42 patients, 1–2000 IU/mL in 45 patients, and > 2000 IU/mL in 13 patients. In addition, 5 HBsAg-positive patients were newly diagnosed during COVID-19 follow-up and were started on treatment for chronic hepatitis B. Of the study cohort, 84.2% were managed in the medical ward and 15.8% were treated in the intensive care unit.

Corticosteroid therapy (methylprednisolone) was administered to 75 patients (51.7%). The median cumulative dose was 520 mg (IQR 240–1400 mg), with a median duration of 6 days (IQR 4–10).

### 4.1. Mortality

**Figure 1.**
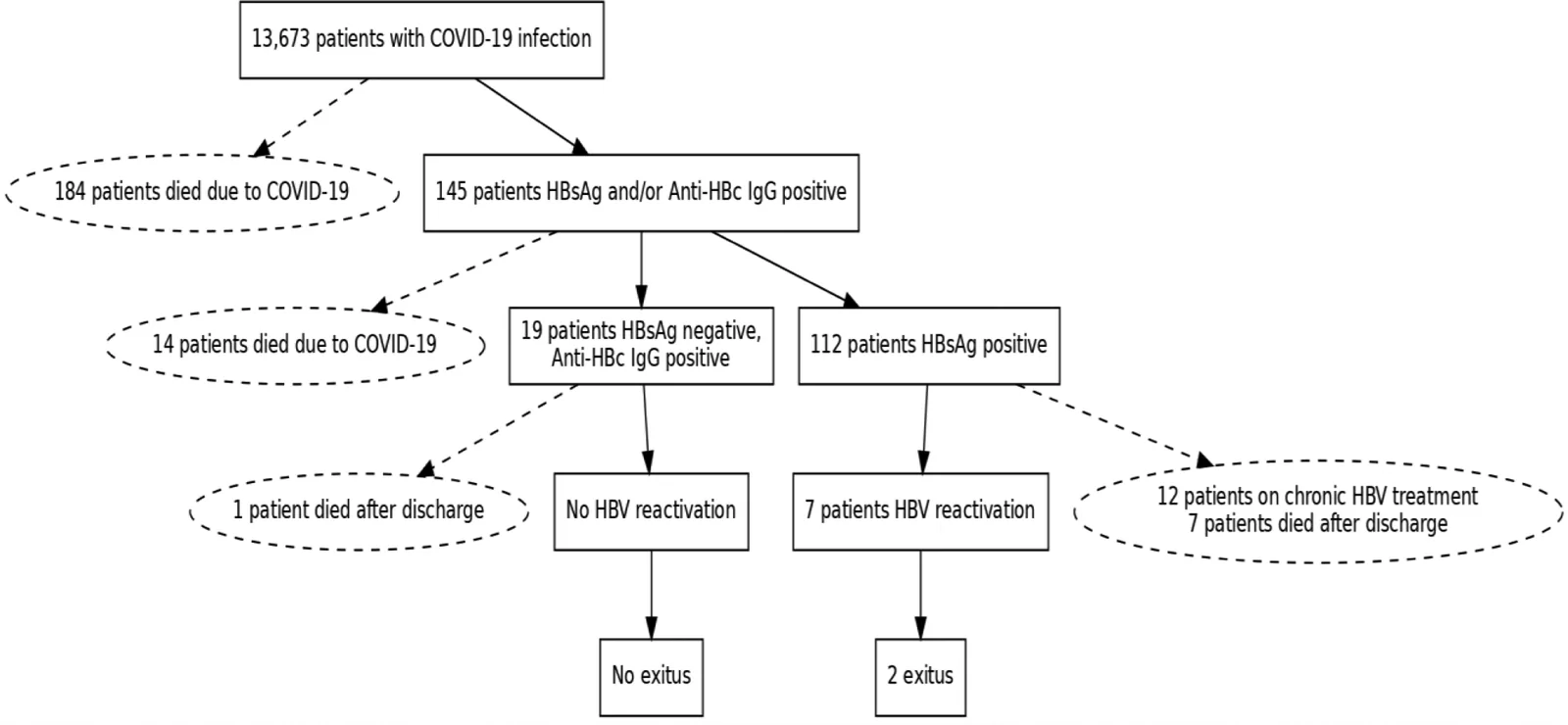
Flow chart of the study population showing screening, enrollment, and clinical outcomes of COVID-19 patients with HBV exposure.

During the study period, 13,673 patients were hospitalized with COVID-19, of whom 184 (1.34%) died. Among the 145 patients with HBV exposure included in this study, 24 (16.6%) died during follow-up. Fourteen patients (9.6%) died from COVID-19–related respiratory failure during hospitalization, while 10 patients died of non-COVID-19 causes after discharge. The patient flow chart is given in Figure 1. Accordingly, the mortality rate among patients with HBV co-infection was substantially higher compared with the overall COVID-19 population (9.6% vs 1.3%).

### 4.2. HBV reactivation

HBV-DNA follow-up was available for 111 patients, including 101 HBsAg-positive and 10 anti-HBc IgG-positive patients. Overall, HBV reactivation occurred in 7 of 145 patients (4.8%) in the total cohort. By serological subgroup, reactivation developed in 7 of 101 HBsAg-positive patients (6.9%), whereas no reactivation was detected among anti-HBc IgG-positive patients (0/10). No serological reactivation was observed in the HBsAg-negative patient group. All patients with reactivation were HBsAg-positive, HBeAg-negative, and anti-HBe-positive, and none of them had used nucleos(t)ide analogues before HBVr. Additionally, 12 HBsAg-positive patients were receiving ongoing chronic nucleos(t)ide analogue therapy prior to baseline; none of these patients experienced virological breakthrough or HBV reactivation during follow-up. The median value of HBV-DNA measured before reactivation was 1834.5 IU/ml. Hepatitis flare was observed in 4 patients, and 2 patients with HBVr died from hepatic failure.

Patients who developed HBVr had received corticosteroids for a significantly longer duration compared to those without reactivation (median 8 [2–25] vs 0 [0–6] days, *P* = .007). In addition, the cumulative methylprednisolone dose was markedly higher in the reactivation group (median 440 [80–2620] vs 0 [0–400] mg, *P* = .021), indicating that both prolonged use and higher doses were associated with an increased risk of reactivation.

### 4.3. Antiviral therapy

Of the seven patients with HBVr, five received nucleos(t)ide analogue treatment (entecavir or tenofovir) with subsequent viral suppression, while two patients developed fatal hepatic failure despite therapy. The distribution of patients with reactivation, according to the pre-and post laboratory values and the reactivation criteria of the guidelines, are given in Table 2.

**Table 2:** Clinical characteristics of patients with HBV reactivation.

Patient	Age/Sex	Comorbidities	Baseline HBV-DNA (IU/mL)	HBV-DNA at Reactivation (IU/mL)	ALT at Reactivation (IU/L)	Steroid Duration (days)	Total Dose (mg)	Hepatitis Flare	Antiviral Therapy	Outcome
1	64/M	COPD	1.02 × 10^6^	1.75 × 10^7^	155	29	2620	Yes	TDF	Death
2	81/M	DM HT	Unknown	5.83 × 10^5^	279	11	640	Yes	ETV	Death
3	39/F	HT HL	2.25 × 10^5^	1.26 × 10^8^	197	2	80	Yes	ETV	Alive
4	49/M	-	1905	2.31 × 10^8^	881	25	6800	Yes	TDF	Alive
5	48/M	-	1764	42,740	49	7	440	No	TDF	Alive
6	56/F	HT	44	668	22	2	80	No	None	Alive
7	69/F	DM HT HL	0	39	17	8	400	No	None	Alive

COPD = Chronic obstructive pulmonary disease, DM = Diabetes Mellitus, ETV = Entecavir, HL = Hyperlipidemia, HT = Hypertension, TDF = Tenofovir disoproxil fumarate.

Among the seven patients with HBVr, two developed fatal hepatic failure. The first was an 81-year-old male who experienced hepatitis exacerbation 4 months after COVID-19 hospitalization, with HBV-DNA rising to 5.83 × 10^5^ IU/mL and progressive liver enzyme elevation, resulting in death due to hepatic failure. The second was a 64-year-old male with COPD who had received methylprednisolone for a total of 29 days, including 5 days of 250 mg pulse therapy, with a cumulative dose of 2620 mg. One month after hospitalization, HBV-DNA reached 1.75 × 10^7^ IU/mL and ALT increased to 155 IU/L, and the patient also died of hepatic failure.

## 5. Discussion

This study investigated the incidence and clinical impact of hepatitis B virus reactivation (HBVr) among patients with COVID-19 and concurrent HBV infection who received corticosteroid therapy. Our findings demonstrate that HBVr can occur even after short courses of corticosteroid treatment, particularly in HBsAg-positive individuals. Among 13,673 hospitalized COVID-19 patients, 145 had HBV exposure, and seven (4.8%) experienced HBV reactivation. Notably, two patients developed hepatic failure and died, highlighting the potential severity of this complication. These results emphasize the clinical importance of HBV screening and close virological monitoring in COVID-19 patients who require corticosteroid therapy.

The observed HBVr rate in our study (4.8%) is comparable to or slightly higher than rates reported in prior non-COVID immunosuppressive settings, suggesting that SARS-CoV-2 infection itself, combined with corticosteroid use, may exacerbate viral reactivation risk.^[7,11,12]^ Previous studies have documented HBVr following chemotherapy and biologic or immunosuppressive therapy, but data on COVID-19–related cases remain limited.^[7–9]^ The reactivations observed in our cohort all occurred in HBsAg-positive patients, consistent with reports indicating that active HBV carriers have a higher susceptibility to viral rebound under immunosuppression. This supports the notion that even transient immunosuppression during COVID-19 management may be sufficient to trigger HBV replication, especially in those with baseline viral persistence. The baseline HBV-DNA distribution of the HBsAg-positive cohort also showed substantial virological heterogeneity, including a subgroup with HBV-DNA > 2000 IU/mL, which is relevant for individualized risk stratification during corticosteroid exposure.

Our findings indicate that both the cumulative dose and duration of corticosteroid therapy contribute to the risk of HBV reactivation (HBVr); nevertheless, reactivation may occur even after short-term, high-dose administration. This observation challenges the conventional four-week threshold currently used to define clinically significant steroid-induced immunosuppression. In regions with intermediate-to-high HBV prevalence, routine HBV screening before initiating corticosteroid therapy is essential, and prophylactic antiviral therapy should be considered for HBsAg-positive patients using high doses of steroids, regardless of treatment duration. Moreover, clinicians should remain vigilant for hepatic flare or viral rebound in patients who develop unexplained liver enzyme elevations during or after COVID-19 therapy. Integration of proactive HBV management – including screening and timely antiviral initiation – into COVID-19 treatment algorithms could substantially reduce preventable hepatic complications and mortality.

Our study demonstrates that co-infection with COVID-19 and HBV may worsen clinical outcomes, with a mortality rate of 9.6% among HBV-infected patients compared to 1.3% in the overall COVID-19 cohort. A recent study also showed that COVID-19 patients with preexisting liver disease had a higher mortality rate compared to those without liver disease.^[13]^ Similarly, several reports indicate that COVID-19 infection is more severe or associated with higher mortality in patients with chronic hepatitis B.^[14–16]^

Corticosteroids remain an effective therapy for severe COVID-19 and may potentially trigger HBV reactivation; however, evidence regarding this risk – particularly with short-term or pulse steroid regimens – remains limited.^[2]^ The mechanisms underlying this effect during prolonged corticosteroid exposure include both direct stimulation of HBV replication and suppression of cytotoxic T-cell function; however, the exact mechanism by which pulse steroid therapy may induce HBV reactivation remains unclear.^[7]^ The risk of reactivation is dose- and duration-dependent, and international guidelines generally define ≥ 20 mg prednisolone-equivalent daily for > 4 weeks as clinically significant immunosuppression.^[8]^ However, our findings – and those of Wong et al – indicate that even shorter courses of high-dose corticosteroids may increase the risk of hepatitis flares, highlighting the need to reconsider the traditional four-week threshold for risk stratification.^[17]^

In our cohort, HBVr was significantly associated with higher cumulative steroid doses and longer duration of use. Importantly, reactivation was observed in patients who had received high-dose corticosteroids for < 4 weeks, suggesting that antiviral prophylaxis should be considered in HBsAg-positive patients receiving high-dose regimens, regardless of treatment duration. This aligns with recent studies recommending prophylaxis for patients receiving pulse steroids or other intensive immunosuppressive therapies.^[17–19]^ A Spanish study of 72 patients with HBV exposure receiving high-dose corticosteroids found no reactivation among patients on nucleos(t)ide prophylaxis, but 1 case occurred in an HBsAg-negative/anti-HBc-positive patient without prophylaxis.^[20]^ In our study, none of the patients received HBV prophylaxis, and all of the reactivations were detected in the HBsAg-positive group.

Several case reports further support the association between COVID-19 and HBVr.^[19,21]^ Reactivation has been described not only in patients treated with systemic corticosteroids but also in those with inhaled corticosteroid use or even without immunosuppressive therapy, likely related to SARS-CoV-2–induced immune dysregulation and severe lymphopenia.^[21]^ In our study, 1 patient developed HBVr after only 2 days of steroid therapy, but with a high baseline HBV-DNA, again highlighting the interplay between host immunity, viral replication, and steroid exposure.

International guidelines (APASL, AGA, AASLD) consistently recommend universal HBV screening prior to initiating immunosuppressive therapy and prophylactic nucleos(t)ide analogue therapy in patients at moderate to high risk of reactivation.^[7,9,10]^ Although our cohort would be classified as low risk according to these criteria, we observed a higher-than-expected rate of HBVr. This suggests that current guideline-based risk stratification may underestimate reactivation risk in certain clinical contexts, such as short-term, high-dose corticosteroid therapy for COVID-19. Despite initiating antiviral therapy after HBVr, two patients in our study died of hepatic failure, emphasizing that prophylaxis is superior to rescue therapy.

Taken together, our results and prior evidence suggest that COVID-19 patients with chronic HBV infection represent a vulnerable group in whom corticosteroid therapy can trigger severe and potentially fatal reactivation.

## 6. Limitations of the study

Our study has several limitations. Patient management during the pandemic was heterogeneous, with variations in screening, monitoring, and corticosteroid protocols across departments. Not all HBsAg-positive patients had HBV-DNA measured, and some were lost to follow-up, which may have led to underestimation of the true incidence of HBVr. Finally, because only 7 HBV reactivation cases were observed, multivariable analysis was not feasible. Accordingly, the associations observed between HBV reactivation and corticosteroid exposure are based on unadjusted comparisons and should be interpreted as hypothesis-generating rather than evidence of independent effect. Despite these limitations, our findings contribute valuable real-world evidence to a poorly studied area.

## 7. Conclusions

COVID-19 patients with chronic HBV infection, particularly those receiving high-dose corticosteroids, are at increased risk of HBVr and poor outcomes. Based on our findings, HBV screening and consideration of antiviral prophylaxis should be routine prior to corticosteroid therapy, particularly in regions with intermediate-to-high HBV prevalence, since HBV reactivation may occur even with short-term corticosteroid exposure.

## Author Contributions

**Conceptualization:** Hatun Öztürk Çerik, Bilge Katirci.

**Data curation:** Hatun Öztürk Çerik, Özlem Aldemir, Bilge Katirci, Burcu Demirhan Kapicioğlu.

**Investigation:** Hatun Öztürk Çerik, Özlem Aldemir, Bilge Katirci, Zeynep Banu Ramazanoğlu.

**Methodology:** Hatun Öztürk Çerik, Bilge Katirci, Burcu Demirhan Kapicioğlu.

**Resources:** Hatun Öztürk Çerik, Burcu Demirhan Kapicioğlu, Zeynep Banu Ramazanoğlu.

**Software:** Hatun Öztürk Çerik, Özlem Aldemir, Burcu Demirhan Kapicioğlu.

**Supervision:** Hatun Öztürk Çerik, Arzu Altunçekiç Yildirim.

**Writing – original draft:** Hatun Öztürk Çerik, Zeynep Banu Ramazanoğlu.

**Formal analysis:** Özlem Aldemir.

**Visualization:** Arzu Altunçekiç Yildirim.

**Writing – review & editing:** Arzu Altunçekiç Yildirim.
